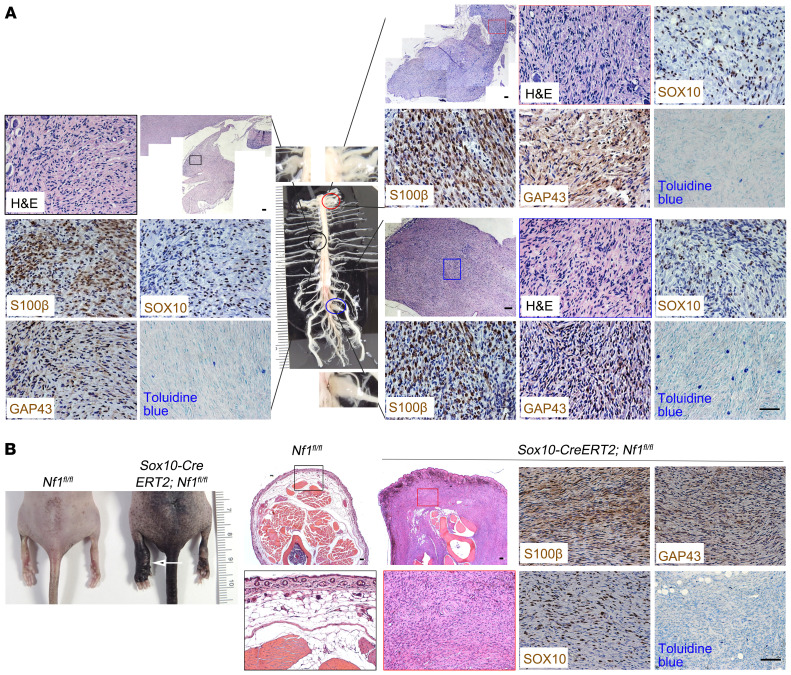# Corrigendum for Humanized neurofibroma model from induced pluripotent stem cells delineates tumor pathogenesis and developmental origins

**DOI:** 10.1172/JCI199700

**Published:** 2025-10-15

**Authors:** Juan Mo, Corina Anastasaki, Zhiguo Chen, Tracey Shipman, Jason Papke, Kevin Yin, David H. Gutmann, Lu Q. Le

Original citation: *J Clin Invest*. 2021;131(1):e139807. https://doi.org/10.1172/JCI139807

Citation for this corrigendum: *J Clin Invest*. 2025;135(20):e199700. https://doi.org/10.1172/JCI199700

In Figure 3A of the original article, the GAP43 image was incorrect and was an inadvertent duplication of the Ku80 image in 3A. In Figure 7A of the original article, the GAP43 image was incorrect and was an inadvertent duplication of the SOX10 image in 7A. Additionally, in Supplemental Figure 3B, the Iba1 image was incorrect and was an inadvertent duplication of the SMA image in Supplemental Figure 3B. The corrected figure panels, based on the original source data, are provided below. The HTML and PDF versions of the paper have been updated and the supplemental file has also been corrected.

The authors regret the errors.

## Supplementary Material

Supplemental data

## Figures and Tables

**Figure 3A:**
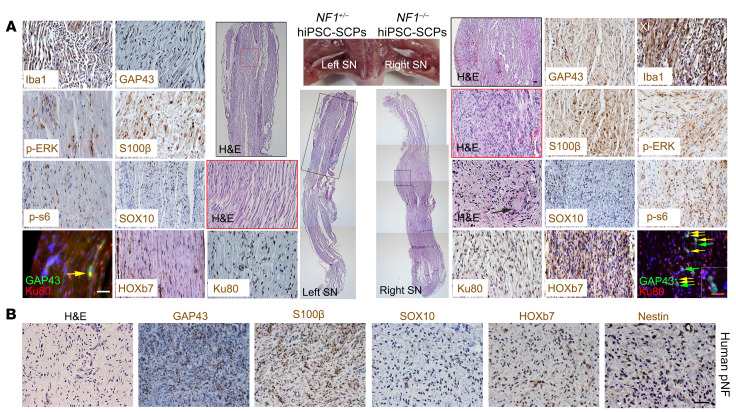


**Figure 7A:**