# Supplementary material for: Corrigendum for Humanized neurofibroma model from induced pluripotent stem cells delineates tumor pathogenesis and developmental origins
Source: J Clin Invest. 2025 Oct 15;135(20):e199700. doi: 10.1172/JCI199700 (PMC12520665; doi:10.1172/JCI199700)
Supplement: Supplemental data [file jci-135-199700-s240.pdf]

Revised Supplemental Figures and Tables for:

Humanized Neurofibroma Model from Induced Pluripotent Stem Cells Delineates Tumor Pathogenesis and Developmental Origins

Juan Mo[1], Corina Anastasaki[2], Zhiguo Chen[1], Tracey Shipman[1], Jason Papke[2], Kevin Yin[1], David H. Gutmann[2,5], Lu Q. Le[1,3,4,5]

[1]Department of Dermatology, UT Southwestern Medical Center, Dallas, TX, USA
[2]Department of Neurology, Washington University School of Medicine, St. Louis, MO, USA
[3]Simmons Comprehensive Cancer Center, UT Southwestern Medical Center, Dallas, TX, USA
[4]Hamon Center for Regenerative Science and Medicine, UT Southwestern Medical Center, Dallas, TX, USA

[5]Corresponding authors

Running title: Humanized Neurofibromas Reveal Tumor Pathogenesis & Origin

Conflict of interest: The authors have declared that no conflict of interest exists.

Keywords
Human iPSC, Neurofibromatosis type 1 (NF1), plexiform neurofibroma (pNF), cutaneous neurofibroma (cNF), Schwann cell precursors (SCP), SOX10, malignant peripheral nerve sheath tumor (MPNST)

Supplemental Figure 1

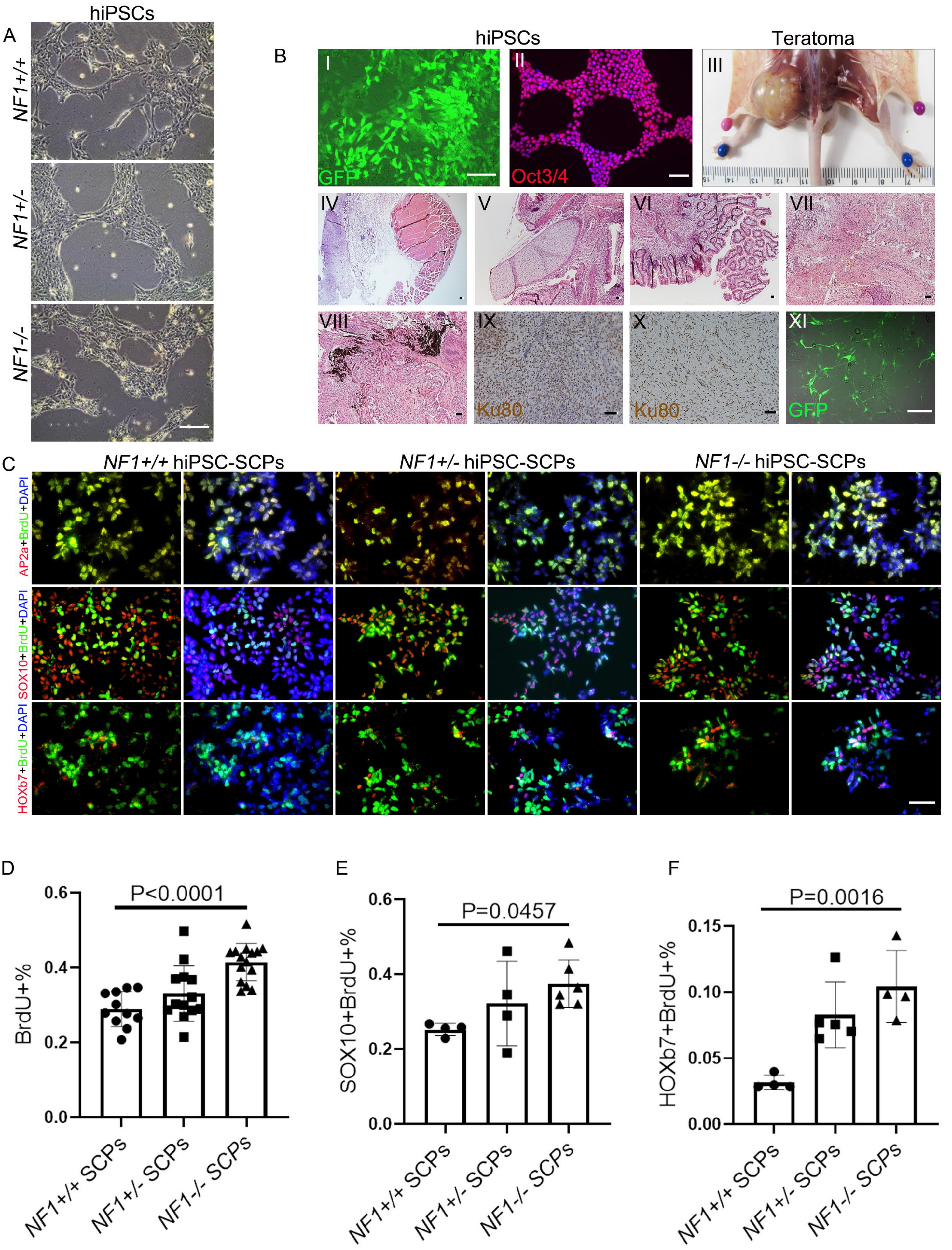

Supplemental Figure 1. Characterization of a series of isogenic hiPSCs and differentiated SCPs that harbor mutations observed in patients with NF1 (related to Figure 1). (A) Bright field images of *NF1+/+*, *NF1+/-*, and *NF1-/-* hiPSCs. **(B)** GFP-tagged hiPSCs (I and II) were injected into athymic mice. (II is a lower magnification image of the Oct3/4 image shown in the Fig. 1A *NF1+/-*hiPSCs Oct3/4 DAPI panel.) The representative teratoma (III) was characterized by H&E and the human specific antibody Ku80. The formation of fat tissue (IV), cartilage (V), luminal-like structures (VI), blood vessels (VII), pigmentation (VIII) and positive staining for Ku80 (IX was teratoma and X was human tissue as positive control) confirmed the pluripotency of hiPSCs. The cells isolated from teratoma (XI) were positive for GFP (XI). n = 3. Scale bar, 50 μm. **(C)** After incubation with BrdU, hiPSC-SCPs were double stained for BrdU and SCP markers including AP2α, SOX10, and HOXb7. Scale bar, 50 μm. **(D)** The percentage of BrdU, SOX10+BrdU+, and HOXb7+BrdU+ cells was calculated and compared between *NF1+/+*, *NF1+/-*, and *NF1-/-* hiPSC-SCPs. Comparisons among groups were performed by one-way ANOVA.

Supplemental Figure 2

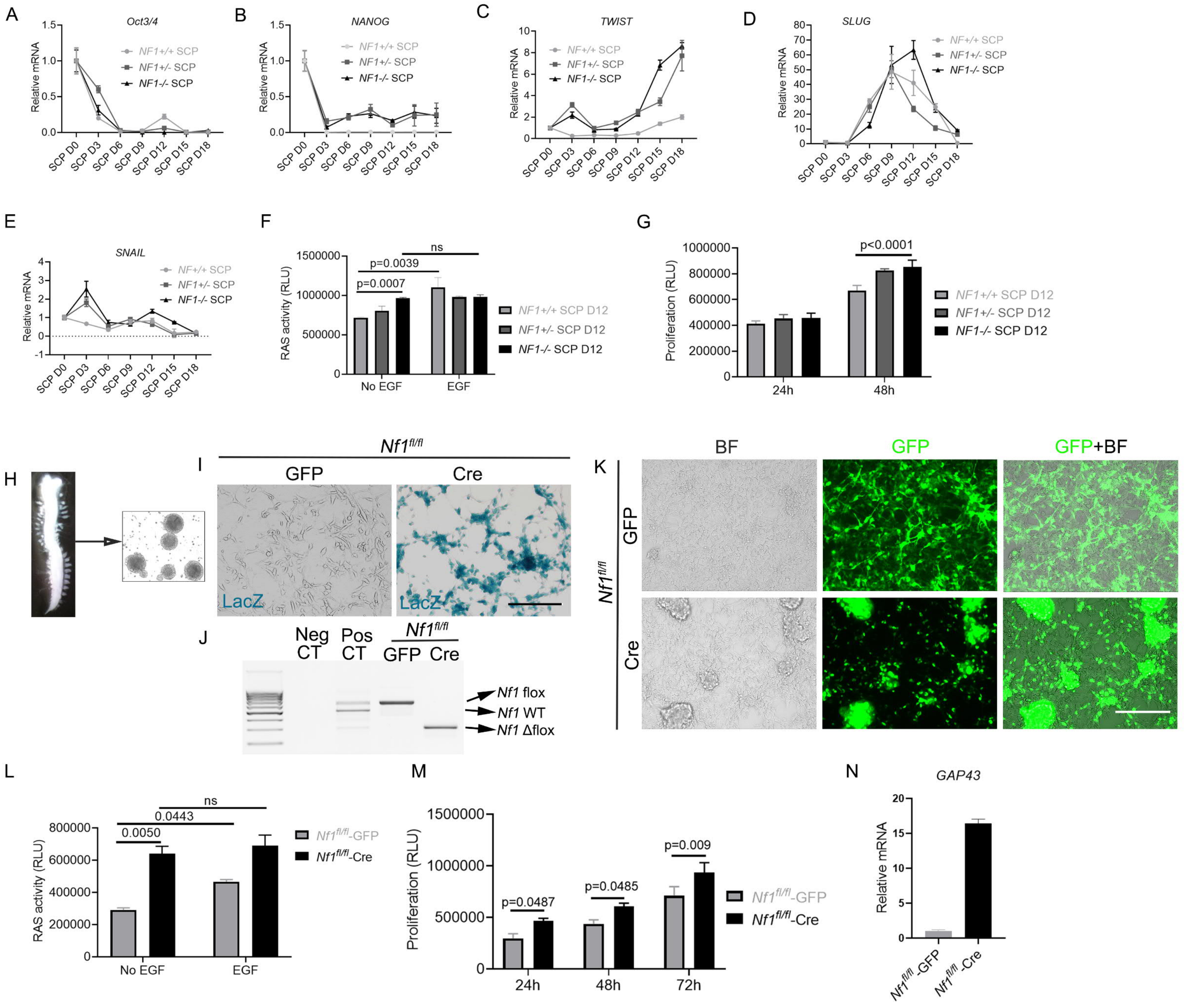

Supplemental Figure 2. *NF1* loss impairs Schwann cell differentiation by maintaining stemness (related to Figure 2). (A-B) mRNA levels of *Oct3/4* and *NANOG* in *NF1+/+*, *NF1+/-*, and *NF1-/-* hiPSC-SCPs were measured. **(C-E)** mRNA levels for migrating NCSCs (*TWIST*, *SLUG*, and *SNAIL*) in *NF1+/+*, *NF1+/-*, and *NF1-/-* hiPSC-SCPs were compared. **(F)** RAS activity was compared between isogenic hiPSC-SCPs with or without EGF. Comparisons between groups were performed by two-way ANOVA. **(G)** Cell proliferation was compared between isogenic hiPSC-SCPs using the cell titer glow assay. Comparisons among groups were performed by one-way ANOVA. **(H)** DNSCs were isolated from E13.5 embryos and formed neurospheres in suspension culture. **(I)** After adenovirus infection, cells were stained with X-gal. The $LacZ^+$ staining in adeno-Cre virus-infected E13.5 $Nf1^{fl/fl}$ DNSCs confirmed *Nf1* deletion. **(J)** *Nf1* deletion was demonstrated by PCR. **(K)** GFP expression was compared between GFP and Cre adenovirus-infected E13.5 $Nf1^{fl/fl}$ DNSCs. **(L)** RAS activity was compared between GFP and Cre adenovirus-infected E13.5 $Nf1^{f/f}$ DNSCs with or without EGF. **(M)** Cell proliferation was compared between GFP and Cre adenovirus-infected E13.5 $NF1^{fl/fl}$ DNSCs using the cell titer glow assay. Comparisons between groups were performed by two-way ANOVA. **(N)** mRNA levels of *GAP43* were compared between GFP and Cre adenovirus-infected E13.5 $Nf1^{f/f}$ DNSCs. Comparisons among groups were performed by one-way ANOVA. Scale bar, 50 μm.

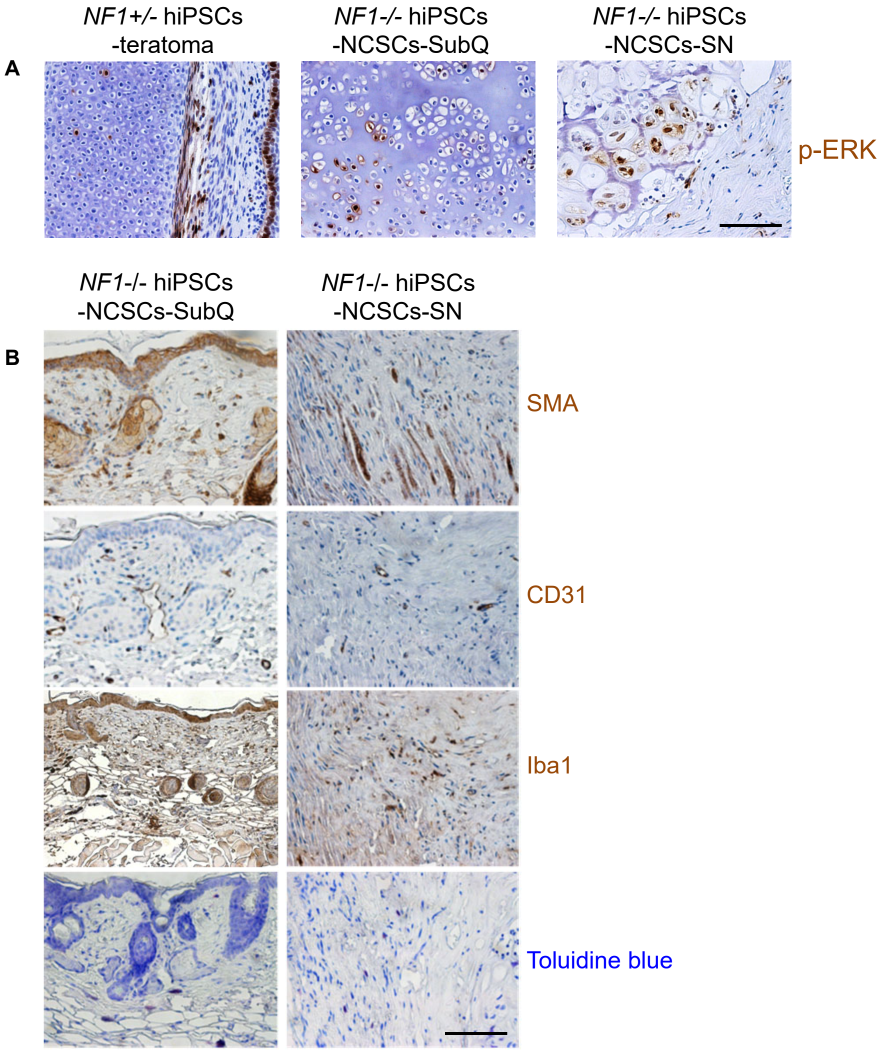

Supplemental Figure 3. Characterization of neurofibroma and cartilage formation (related to Figure 4). (A) Immunostaining of the indicated tissue injected with hiPSCs or hiPSC-NCSCs for expression of phospho-ERK (p-ERK). SubQ = subcutaneous; SN = sciatic nerve. (B) Immunostaining of the indicated tissue injected with *NF1-/-* hiPSC-NCSCs for expression of SMA (fibroblasts), CD31 (endothelial cells), Iba1 (macrophages), and Toluidine blue (mast cells). n = 3. Scale bar, 50 µm.

Supplemental Figure 3. Characterization of neurofibroma and cartilage formation (related to Figure 4). (A) Immunostaining of the indicated tissue injected with hiPSCs or hiPSC-NCSCs for expression of phospho-ERK (p-ERK). SubQ = subcutaneous; SN = sciatic nerve. **(B)** Immunostaining of the indicated tissue injected with *NF1-/-* hiPSC-NCSCs for expression of SMA (fibroblasts), CD31 (endothelial cells), Iba1 (macrophages), and Toluidine Blue (mast cells). n = 3. Scale bar, 50 μm.

Supplemental Figure 4

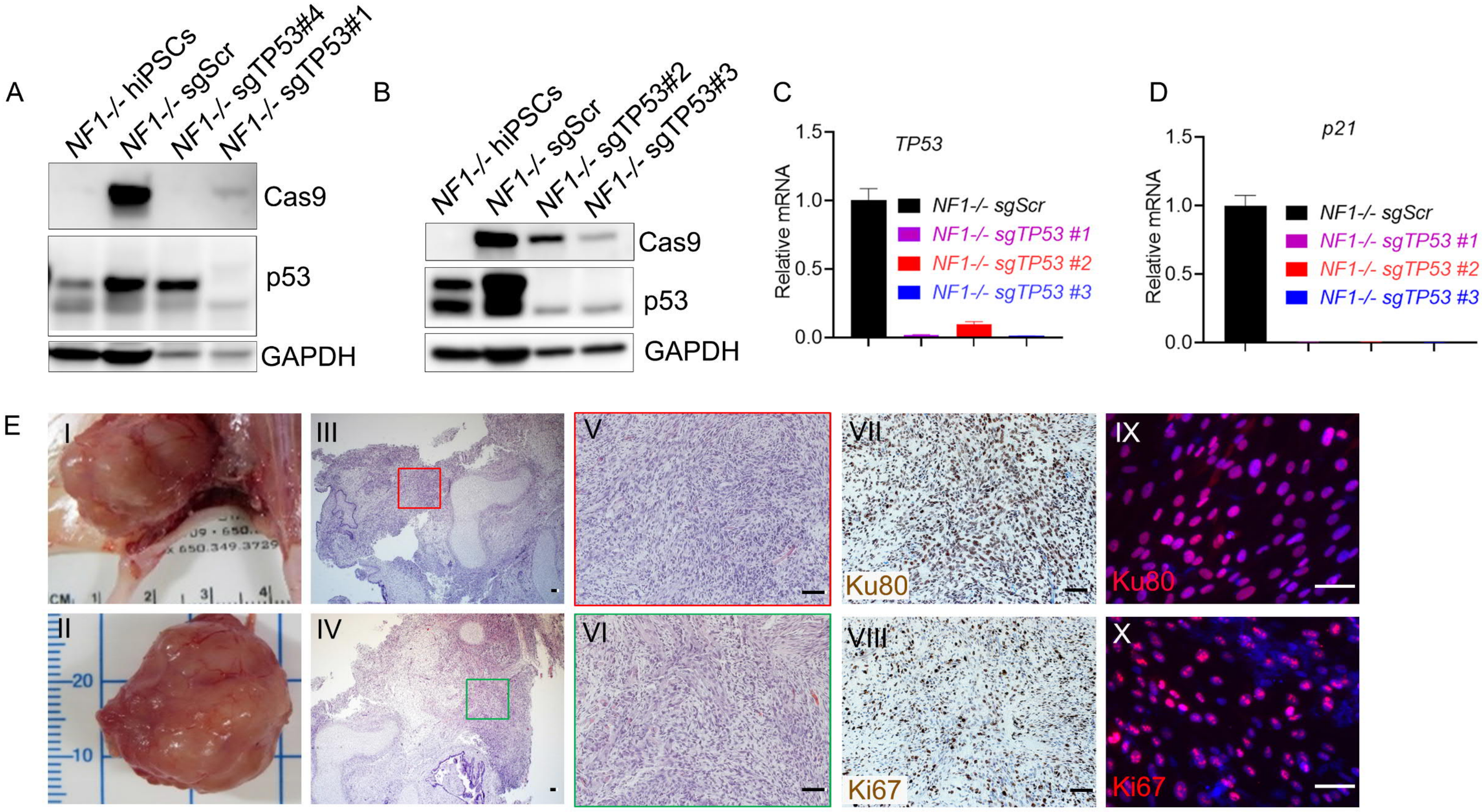

Supplemental Figure 4. Generation and characterization of *NF1-/- sgTP53* hiPSCs (related to Figure 5). (A) Expression of Cas9 and p53 were measured by Western blot. GAPDH was used as a loading control. *NF1-/-* sgTP53#4 failed because Cas9 was not successfully transfected. *TP53* deletion in *NF1-/-* sgTP53#1 was confirmed. **(B)** Expression of Cas9 and p53 were measured by Western blot. GAPDH was used as an internal loading control. *TP53* deletion in *NF1-/-* sgTP53#2 and #3 was confirmed. (Same blot as shown in Fig. 5B, but also showing the additional lane with *NF1-/-* sgTP53#3.) **(C-D)** mRNA levels of *TP53* and *p21* were measured before and after CRISPR/Cas9 engineered *TP53* deletion. **(E)** CRISPR/Cas9 engineered *NF1-/- sgTP53* hiPSCs were injected into athymic mice. Cartilage (III and IV), luminal-like structures (III and IV) and hypercellularity (V and VI) were observed in representative teratomas (I and II), which were positive for Ku80 (VII and IX) and Ki67 (VIII and X). n = 3. Scale bar, 50 µm.

Supplemental Figure 5

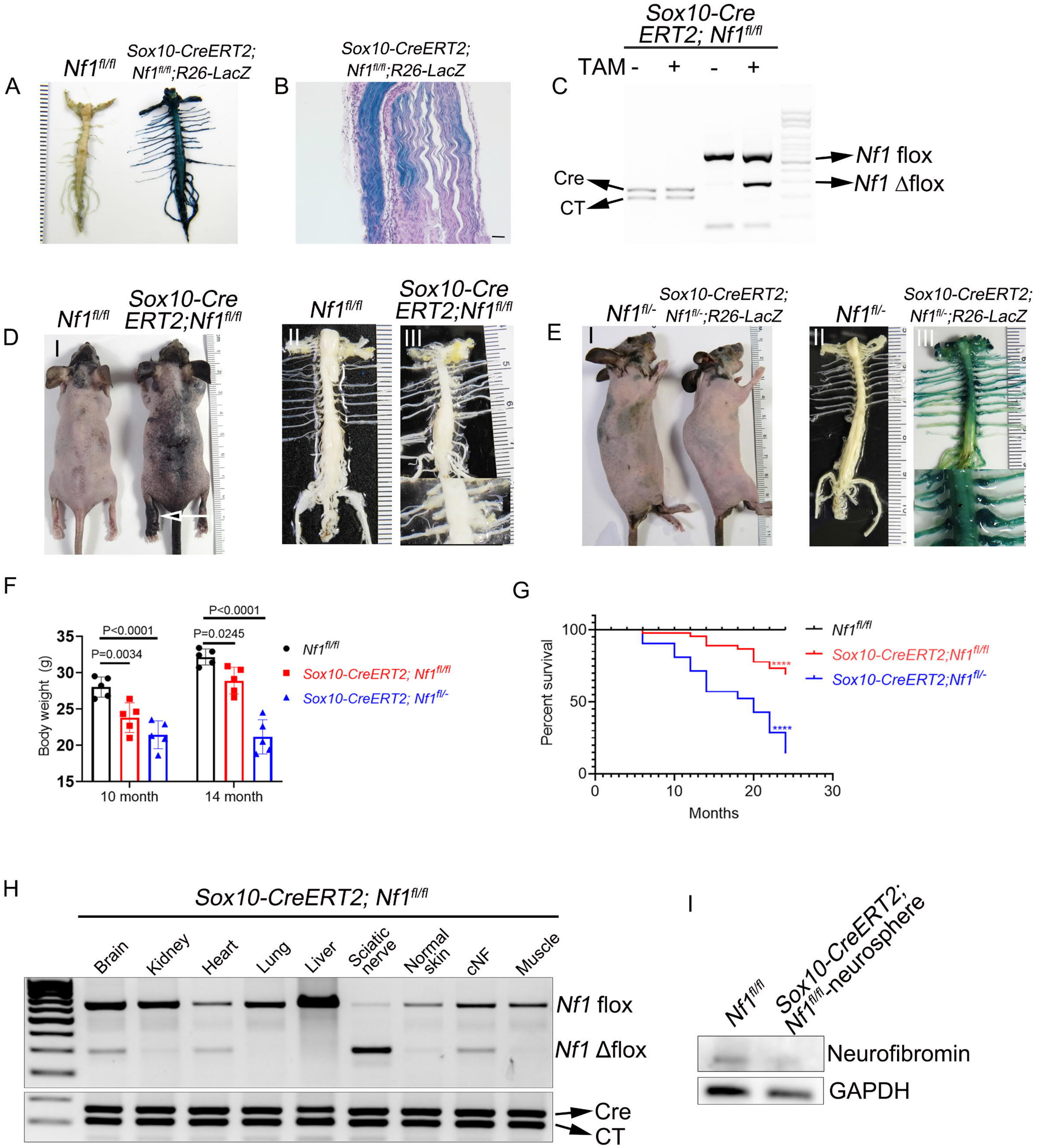

Supplemental Figure 5. Characterization of *Sox10-CreERT2; Nf1$^{fl/fl}$* mice (related to Figure 7). (A-B) After tamoxifen treatment, LacZ was expressed in the spinal cord (**A**) and sciatic nerve (**B**). **(C)** *Nf1* deletion between two LoxP sites in *Sox10-CreERT2; Nf1$^{fl/fl}$* mice after tamoxifen administration was confirmed by demonstration of a Δflox band. **(D)** A representative *Sox10-CreERT2; Nf1$^{fl/fl}$* mouse with a classic giant diffuse plexiform neurofibroma (arrow) after tamoxifen treatment exhibits a giant thigh/leg tumor and enlarged DRGs (Insert in III). This representative plexiform neurofibroma on thigh/leg in D-I was from the same mouse that is shown in Figure 7B. **(E)** A representative *Sox10-CreERT2; Nf1$^{fl/-}$; R26-LacZ* mouse with a pNF showed spinal cord curve kyphosis and enlarged DRGs (Insert in III). Scale bar, 50 µm. **(F)** Comparison of body weights between *Nf1$^{fl/fl}$*, *Sox10-CreERT2; Nf1$^{fl/fl}$* and *Sox10-CreERT2; Nf1$^{fl/-}$* mice. Comparisons among groups were performed by one-way ANOVA. **(G)** Kaplan-Meier survival curves of *Nf1$^{fl/fl}$*, *Sox10-CreERT2; Nf1$^{fl/fl}$* and *Sox10-CreERT2; Nf1$^{fl/-}$* mice with log-rank statistical test. **(H)** Genomic DNA was isolated from different tissues in *Sox10-CreERT2; Nf1$^{fl/fl}$* mice and genotyping for *Nf1* flox and *Nf1* Δflox was measured by PCR. **(I)** Neurofibromin expression in neurospheres isolated from cNFs of *Sox10-CreERT2; Nf1$^{fl/fl}$* mice was determined by western blot. GAPDH was the loading control.

Supplemental Figure 6

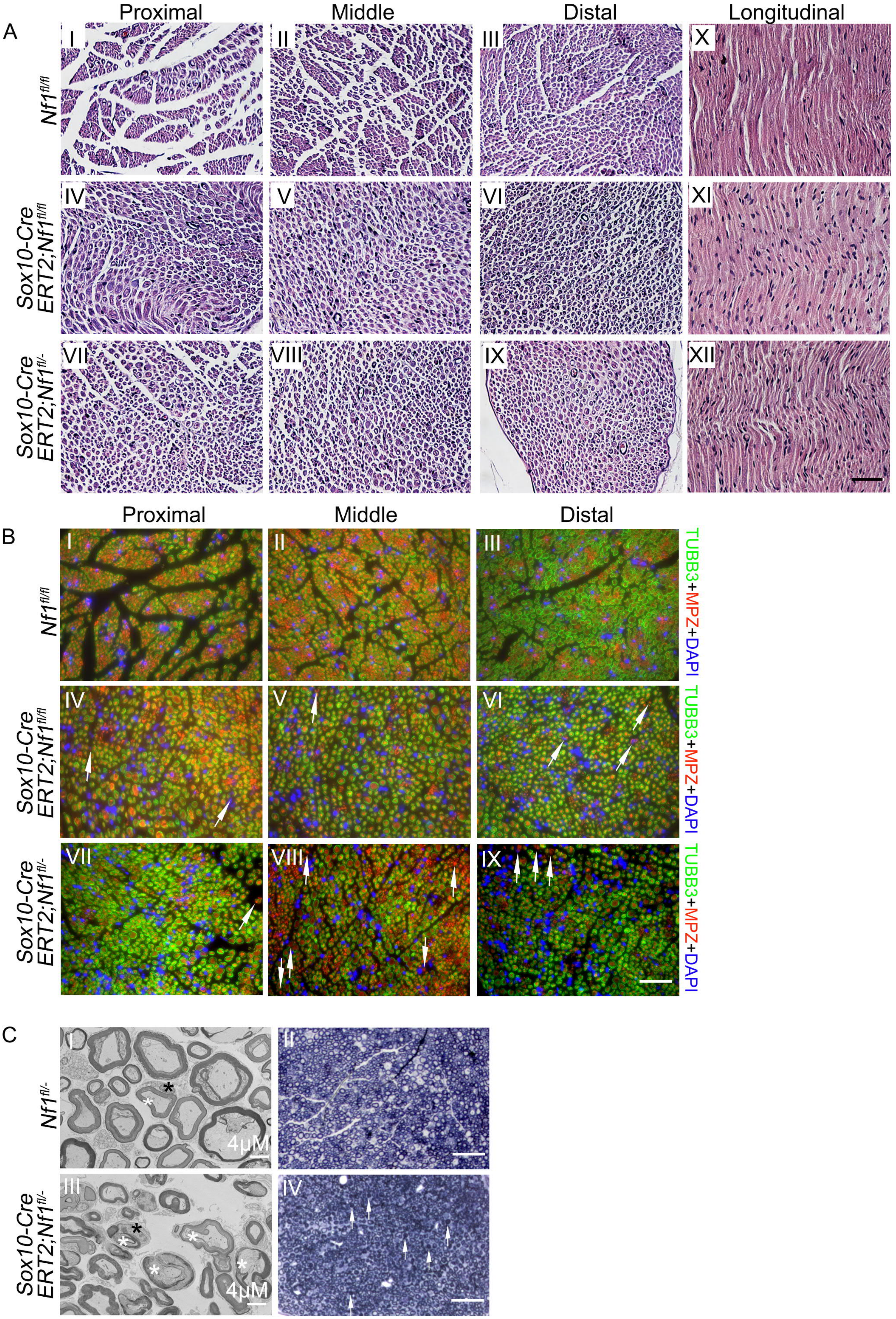

Supplemental Figure 6. *Nf1* loss in SOX10-expressing cells impairs Schwann cell lineage differentiation *in vivo* (related to Figure 7). (A) Sciatic nerves from *Nf1$^{fl/fl}$*, *Sox10-CreERT2; Nf1$^{fl/fl}$* and *Sox10-CreERT2; Nf1$^{fl/-}$* mice were cross-sectioned (I to IX) and longitudinally sectioned (X to XII). H&E showed that mutant sciatic nerves lost the nerve bundles with fascicular organization compared to controls (I to IX). Mutant sciatic nerves were more hypercellular than controls (X to XII). n = 3. **(B)** Mutant sciatic nerves developed more non-myelinated axons (white arrows) compared to controls. n = 3. **(C)** Electron micrographs (I and III) showed that mutant sciatic nerves had non-myelinated and immature sheath surrounding the axon (white star in III). Black star shows a Schwann cell surrounding an axon. Toluidine blue staining (II and IV) showed the increased numbers of cells between myelinated axons in the mutant sciatic nerves (white arrows in IV). Scale bar, 50 μm unless otherwise noted.

Supplemental Figure 7

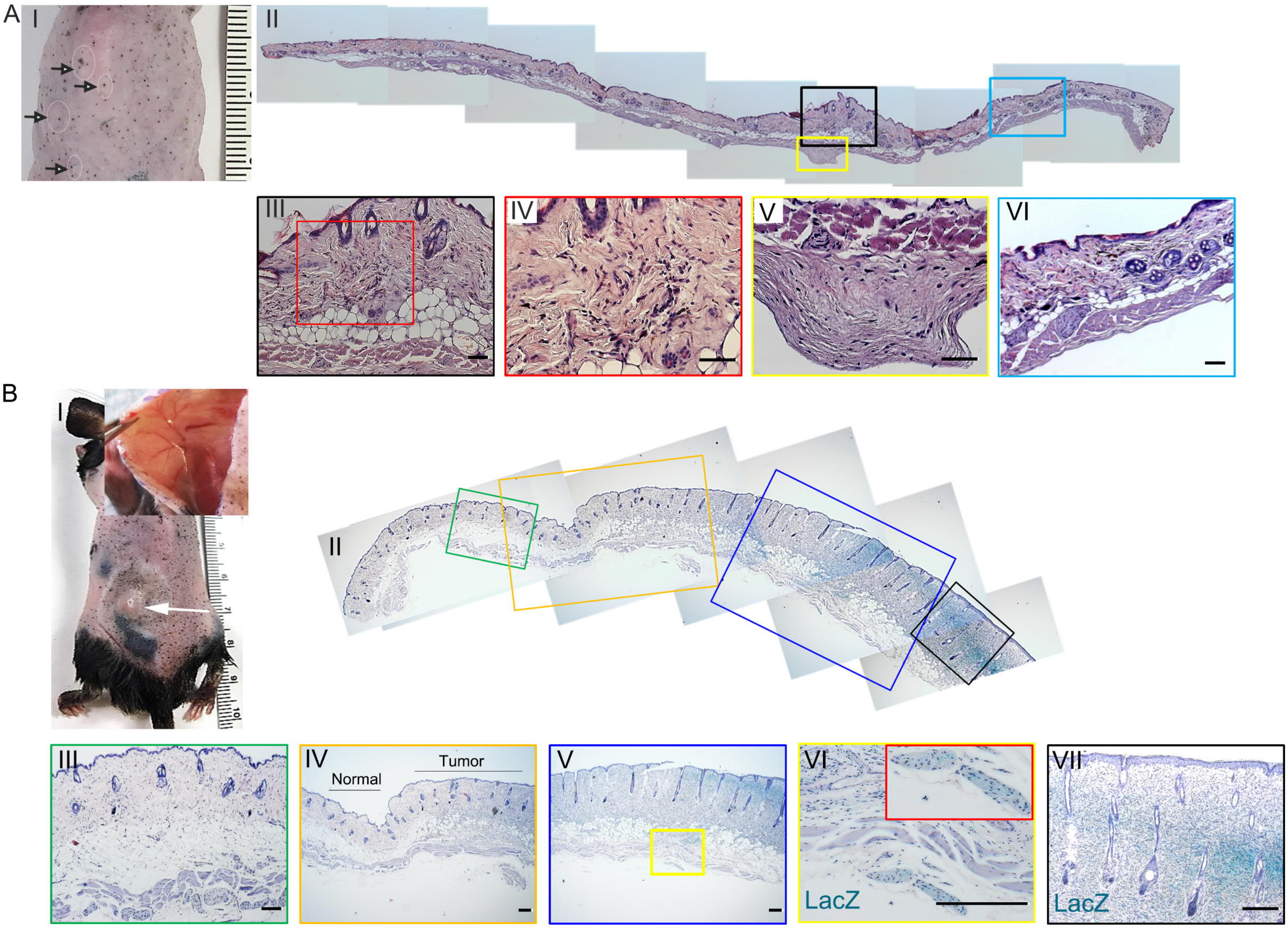

Supplemental Figure 7. SOX10-expressing cells contain the cNF tumor-initiating cells (related to Figure 8). (A) *Sox10-CreERT2; Nf1$^{fl/fl}$; R26-LacZ* mice developed early signs of discrete cutaneous neurofibromas (circles in I and squares in II) following tamoxifen treatment, showing hypercellularity (III and IV) and enlarged nerves (V) compared to the “normal” skin distant from the tumor (VI). **(B)** Upon tamoxifen treatment, *Sox10-CreERT2; Nf1$^{fl/fl}$; R26-LacZ* mice gradually developed discrete cutaneous neurofibromas (I), with a distinct transition from thin skin to thick skin (II - V). The tumor area had accumulation of nerves (VI) and LacZ-positive SOX10 lineage cells (VII). These representative mouse skin pictures with cutaneous neurofibroma in B-I were from the same mouse that is shown in Figure 8A. Scale bar, 50 μm.

Supplemental Figure 8

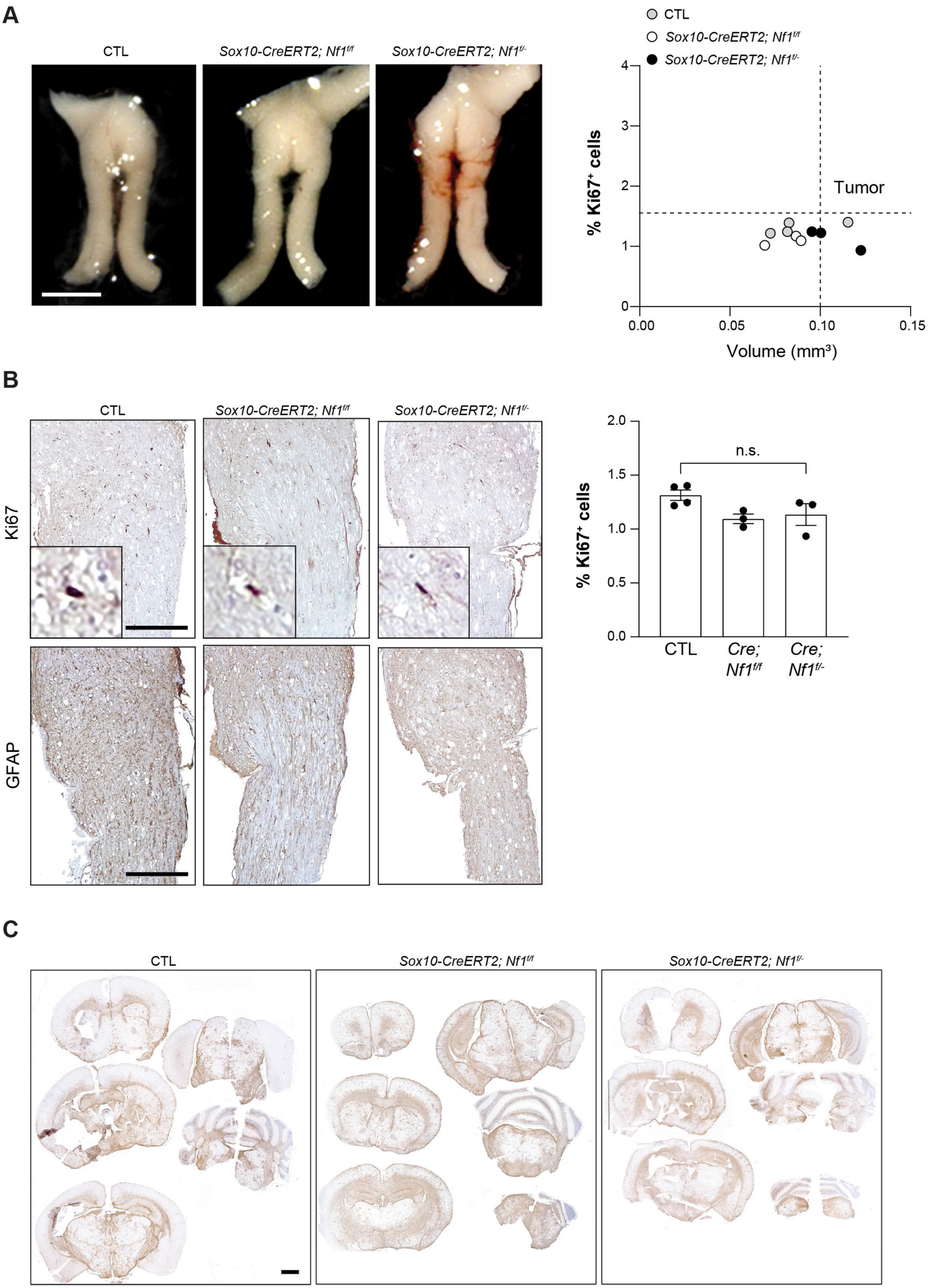

Supplemental Figure 8. *Sox10*-CreERT; *Nf1*$^{fl/-}$ mice do not develop central nervous system tumors. (**A**) Representative gross images of optic nerves (left panel) from *Sox10-CreERT; Nf1*$^{fl/-}$ mice and *Sox10-CreERT; Nf1*$^{fl/fl}$ mice, compared to *Nf1*$^{fl/fl}$ controls (CTL), with prechiasmatic optic nerve volumes plotted against their respective percentage of Ki67^{+} cells (rig*ht panel*). Scale bar, 1 mm. (**B)** Representative Ki67 and GFAP immunostaining of *Sox10-Cre; Nf1*$^{fl/fl}$ and *Sox10-Cre; Nf1*$^{fl/-}$ optic nerves, compared to *Nf1*$^{fl/fl}$ controls**.** Quantitation of the percent of Ki67^{+} cells is shown in the graph (right). Data are presented as the mean ± SEM. One-way ANOVA with Bonferroni post-test correction. n.s., not significant. Scale bar, 100 µm. **(C)** Representative GFAP-immunostained whole brain sections from *Sox10-Cre; Nf1*$^{fl/fl}$ and *Sox10-Cre; Nf1*$^{fl/-}$ mice compared to *Nf1*$^{fl/fl}$ controls**.** No optic gliomas or brain tumors were identified in any of the mutant mice. Scale bar, 100 µm.

Supplemental Table 1. Band intensity ratios (related to Figures 2)

Cell line	p-ERK/ t-ERK	p-ERK/ GAPDH	t-ERK/ GAPDH	GAP43/ GAPDH	SOX10/ GAPDH	p53/ GAPDH	
NF1+/- hiPSCs-SCPs	0.37	0.30	0.79	1.07	0.82	0.99	
NF1-/- hiPSCs-SCPs	0.49	0.60	1.23	1.47	0.69	0.77	
	**p-ERK/ t-ERK**	**p-ERK/ GAPDH**	**t-ERK/ GAPDH**	**GAP43/ GAPDH**	**SOX10/ GAPDH**	**p53/ GAPDH**	**Nestin/ GAPDH**
$Nf1^{fl/fl}$-GFP	0.95	0.19	0.20	0.54	0.31	0.23	0.20
$Nf1^{fl/fl}$ -Cre	0.68	0.28	0.41	0.69	0.24	0.21	0.28

Supplemental Table 2. Resources/Chemicals used

Name	Company	Catalog No.
DMEM/F12	Gibco	Cat#12-634-028
Neurobasal medium	Gibco	Cat#21103049
N2	Gibco	Cat#17502-048
B27	Gibco	Cat#12587-010
mTeSR plus medium	STEMCELL Technologies	Cat#5825
StemPro™ Accutase™ Cell Dissociation Reagent	Thermofisher	Cat#A1110501
ROCK inhibitor (Y-27632)	STEMCELL Technologies	Cat#72304
Cryostor CS10	STEMCELL Technologies	Cat#07930
Probumin BSA	EMD Millipore	Cat# 810683
Glutamax	Gibco	Cat#35050-061
Non-essential amino acid	Corning	Cat#25-025-CI
Trace A	Corning	Cat#25-021-CI
Trace B	Corning	Cat#25-022-CI
Trace C	Corning	Cat#25-023-CI
Bovine transferrin	Invitrogen	Cat#11107-018
(+)-sodium l-ascorbate	Sigma	Cat# A4034
LONGR3 IGF-I	Sigma	Cat#85580C
CHIR99021	STEMCELL Technologies	Cat# 72052
Human recombinant FGF2	Thermo Fisher	Cat#PHG0360
Neuregulin β-1	Peprotech	Cat#AF-100-03
SB431542	EMD Millipore	Cat#61-6461-5MG
Y27632 (ROCK inhibitor)	STEMCELL Technologies	Cat#72304
LDN193189	Tocris	Cat# 60-531-0
2-mercaptoethanol	Gibco	Cat#21985-023
L-15 medium	Sigma	Cat#L1518
pMD2.G	Addgene	Plasmid#12259
psPAX2	Addgene	Plasmid#12260
LentiCRISPRv2	Addgene	Plasmid#52961
Ad5CMVGFP	University of Iowa	VVC-U of Iowa-4
Ad5CMVCre-eGFP	University of Iowa	VVC-U of Iowa-1174-HT
iTaq™ Universal SYBR Green Supermix	BIO-RAD	Cat#172-5124
iScript™ Select cDNA Synthesis Kit	BIO-RAD	Cat#1708897
2XTaq RED Master Mix	Apex	Cat#5200300-1250

NucleoBond Xtra Midi	Macherey-Nagel	Cat#740410.100
PD0325901	Selleckchem	Cat#S1036

Supplemental Table 3. Antibodies used

Antibody	Company	Identifier
Mouse monoclonal anti NGFR (p75)	Advanced Targeting Systems	Cat#AB-N07, RRID: AB_171797
Chicken polyclonal anti GAP43	Novus	Cat#NBP1-92714, RRID: AB_11023277
Rabbit polyclonal anti S100β	Agilent	Cat# Z0311 RRID: AB_10013383
Rabbit polyclonal anti GAP43	Abcam	Cat#ab12274, RRID: AB_2247459
Mouse monoclonal anti SOX10 (A-2)	Santa Cruz	Cat#sc-365692, RRID: AB_2721184
Rabbit monoclonal anti SOX10 [EPR4007-104]	Abcam	Cat#ab180862, RRID: AB_2721184
Chicken polyclonal anti nestin	Abcam	Cat#ab134017, RRID: AB_2753197
Rabbit polyclonal anti phospho-H3 (Ser10)	Cell Signaling	Cat#9701, RRID: AB_331535
Rabbit monoclonal anti phospho-ERK1/2	Cell Signaling	Cat#4370, RRID: AB_2315112
Rabbit monoclonal anti total ERK1/2	Cell Signaling	Cat#4695, RRID: AB_390779
Rabbit monoclonal anti phospho-s6	Cell Signaling	Cat#4858 RRID: AB_916156
Rabbit monoclonal anti total s6	Cell Signaling	Cat#2217 RRID: AB_331355
Rabbit polyclonal anti Iba1	Wako	Cat#019-19741 RRID: AB_839504
Rabbit monoclonal anti Ku80	Cell signaling	Cat#2180, RRID: AB_2218736
Mouse monoclonal anti Cas9 (7A9-3A3)	Santa Cruz	Cat#sc-517386, RRID: AB_2800509
Goat polyclonal anti Oct3/4 (N-19)	Santa Cruz	Cat#sc-8628, RRID: AB_653551
Mouse monoclonal anti Oct3/4 (C-10)	Santa Cruz	Cat#sc-5279, RRID: AB_628051
Mouse monoclonal anti TRA1-60	Invitrogen	Cat#41-1000, RRID: AB_2533494
Rabbit polyclonal anti NANOG (N3C3)	GeneTex	Cat#GTX100863, RRID: AB_10615506
Mouse monoclonal anti SOX2	R&D	Cat#MAB2018, RRID: AB_358009
Mouse monoclonal anti HNK1 (CD57)	Sigma	Cat#C6680-50TST, RRID: AB_1078474
Mouse monoclonal anti AP2-alpha	DSHB	Cat#3B5, RRID: AB_528084
Mouse monoclonal anti GAPDH (6c5)	Santa Cruz	Cat#sc-32233, RRID: AB_627679

Rabbit monoclonal anti p53 [26]	Abcam	Cat#ab32389,<br>RRID: AB_776981
Mouse monoclonal anti APC-p75 IgG1 (clone 74902)	R&D	Cat#FAB367A<br>RRID: AB_10920942
Mouse monoclonal anti APC-isotype IgG1 (clone 11711)	R&D	Cat#IC002A<br>RRID: AB_357239
Rabbit polyclonal anti neurofibromin	Santa Cruz	Cat#sc-67,<br>RRID: AB_2149681
Rabbit polyclonal anti HOXb7	Novus	Cat#NBP2-14098,<br>RRID: AB_2721144

Supplemental Table 4. qPCR primers used (related to Figures 2 and 5)

Gene Symbol	Primer Sequence (5'-3')	NCBI RefSeq	Species
actin beta	F: ACCTTCTACAATGAGCTGCG<br>R: CCTGGATAGCAACGTACATGG	NM_001101.3	*Homo sapiens*
CDH19	F: ACAAGCGTCTGTAACTCTGGG<br>R: AGCAAACTTCGTGTTGGACA	NM_021153.4	*Homo sapiens*
ErbB3	F: GGTGCTGGGCTTGCTTTT<br>R: CGTGGCTGGAGTTGGTGTTA	NM_001982	*Homo sapiens*
GAP43	F: AGCCAAGCTGAAGAGAACATAG<br>R: TCAGGCATGTTCTTGGTCAG	NM_002045	*Homo sapiens*
HOXB7	F: CCTGGATGCGAAGCTCAG<br>R: CGTCAGGTAGCGATTGTAGTG	NM_004502.4	*Homo sapiens*
ITGA4	F: AGCCCTAATGGAGAACCTTGT<br>R: CCAGTGGGGAGCTTATTTTCAT	NM_000885.6	*Homo sapiens*
MPZ	F: AAGTGCCAACTAGGTACGGG<br>R: CATAGCACTGAGCCTCCTCT	NM_001315491.1	*Homo sapiens*
nestin	F: CAGCGTTGGAACAGAGGTTA<br>R: GCTGGCACAGGTGTCTCAAG	NM_006617.1	*Homo sapiens*
PAX3	F: GTTTCGCCTTCACCTGGATA<br>R: GTTGATAAAAACACCGCCGA	NM_000438	*Homo sapiens*
PLP1	F: CAGTCTATTGCCTTCCCCAG<br>R: CAGATGGACAGAAGGTTGGAG	NM_000533.5	*Homo sapiens*
p21	F: CATGTGGACCTGTCACTGTCTTGTA<br>R: GAAGATCAGCCGGCGTTTG	NM_001374511.1	*Homo sapiens*
p75NTR	F: CGGCACGTATTCCGACG<br>R: GGTGTGGACCGTGTAATCC	NM_002507.4	*Homo sapiens*
SLUG	F: CGAACTGGACACACATACAGTG<br>R: CTGAGGATCTCTGGTTGTGGT	NM_003068.5	*Homo sapiens*
SNAI1 (SNAIL)	F: TCCACGAGGTGTGACTAACTATGC<br>R: GAATAGTTCTGGGAGACACATCGG	NM_005985.4	*Homo sapiens*
SOX2	F: GCTGCAAAAGAGAACACCAATCCC<br>R: AAACTTCCTGCAAAGCTCCTACCG	NM_003106.4	*Homo sapiens*
SOX10	F: CCTCACAGATCGCCTACACC<br>R: CATATAGGAGAAGGCCGAGTAGA	NM_006941.4	*Homo sapiens*
TP53	F: GCCCAACAACACCAGCTCCT<br>R: GCCCAACAACACCAGCTCCT	NM_001126114.2	*Homo sapiens*
TWIST1	F: GTCCGCAGTCTTACGAGGAG<br>R: GCTTGAGGGTCTGAATCTTGCT	NM_000474.4	*Homo sapiens*

actin beta	F: CCTCTATGCCAACACAGT<br>R: AGCCACCAATCCACACAG	NM_007393.5	*Mus musculus*
Bmi1	F: CTGGAGAAGAAATGGCCCACTA<br>R: CTCATCTTCATTCTTTTGCAAGTTG	NM_007552.4	*Mus musculus*
Ccnd2	F: ACCTCCCGCAGTGTTCCTATTTCA<br>R: AGTCAGCGGGATGGTCTCTTTCAG	NM_009829.3	*Mus musculus*
c-jun	F: CTGAGTGTGCGAGAGACAGC<br>R: CCAAGTCCGTCCGTCTGT	NM_010591.2	*Mus musculus*
Cd133 (prominin-1)	F: GAAGGAGCCCAGCTTAGAGG<br>R: GGTCATTCACTCAAAGTACCATCC	NM_001163577.1	*Mus musculus*
Dhh	F: CATGTGGCCCGGAGTACGCC<br>R: CGCTGCATCAGCGGCCAGTA	NM_007857.5	*Mus musculus*
ErbB3	F: GAGCGGGGTGACGGGAGTAA<br>R: GGGTCGCGAACAGTTCTCCC	NM_010153.2	*Mus musculus*
Ednrb	F: TCAGAAAACAGCCTTCATGC<br>R: GCGGCAAGCAGAAGTAGAAA	NM_001136061.2	*Mus musculus*
Gap43	F: AAGGCAGGGGAAGATACCAC<br>R: TTGTTCAATCTTTTGGTCCTCAT	NM_008083.2	*Mus musculus*
Hey2	F: GTGGGGAGCGAGAACAATTA<br>R: GTTGTCGGTGAATTGGACCT	NM_013904.1	*Mus musculus*
Hmga2	F: AAGGCAGCAAAAACAAGAGC<br>R: CCGTTTTTCTCCAATGGTCT	NM_001347170.1	*Mus musculus*
Hopx	F: CGGAGGACCAGGTGGAGAT<br>R: CCGGGTGCTTGTTGACCTT	NM_001159900.1	*Mus musculus*
Id2	F: GACAGAACCAGGCGTCCA<br>R: AGCTCAGAAGGGAATTCAGATG	NM_010496.3	*Mus musculus*
Igf2bp2	F: GGGAAAATCATGGAAGTTGACTA<br>R: CGGGATGTTCCGAATCTG	NM_183029.2	*Mus musculus*
Krox20 (Egr2)	F: AATGGCTTGGGACTGACTTG<br>R: GCCAGAGAAACCTCCATTCA	NM_010118.3	*Mus musculus*
Krt42	F: AGGAGCTGGCCTATCTGAGG<br>R: GGCGTCCATCTCCACATT	NM_212483.3	*Mus musculus*
Lif	F: AATGCCACCTGTGCCATACG<br>R: CAACTTGGTCTTCTCTGTCCCG	NM_008501.2	*Mus musculus*
Lgr5	F: CAGTGTGGACGACCTTCATAAGAA<br>R: AAGGTCCCGCTCATCTTGAAC	NM_010195.2	*Mus musculus*
Mgll	F: GCAGTGGAACATCTCAACCA<br>R: AGGGAGGCTGTTCCCCTAT	NM_001166251.1	*Mus musculus*
Olfm4	F: TGGCCCTTGGAAGCTGTAGT<br>R: ACCTCCTTGGCCATAGCGAA	NM_001351947.1	*Mus musculus*
p75NTR	F: GGTGATGGCAACCTCTACAGT<br>R: CCTCGTGGGTAAAGGAGTCTA	NM_033217.3	*Mus musculus*
S100β	F: GACTCCAGCAGCAAAGGTGAC<br>R: CATCTTCGTCCAGCGTCTCCA	NM_009115.3	*Mus musculus*
Slug	F: CTGCGGCAAGGCGTTT<br>R: CGTGTGAGTTCTAATGTGTCCTTGA	NM_001168276.1	*Mus musculus*
Sox2	F: GCTGGACTGCGAACTGGA<br>R: GCGTTAATTTGGATGGGATTG	NM_011443.4	*Mus musculus*
Sox10	F: CAGGTGTGGCTCTGCCCACG<br>R: GTGTAGAGGGGCCGCTGGGA	NM_011437.1	*Mus musculus*

Supplemental Table 5. Primers used for CRISPR-Cas9 plasmid (related to Figure 5)

Name	Primer sequence (5'-3')	PAM	NCBI RefSeq
sgTP53-1	F: CACCGGCATGGGCGGCATGAACCGG<br>R: AAACCCGGTTCATGCCGCCCATGCC	GCATGGGCGGCAT<br>GAACCGGAGG	NM_001126114.2
sgTP53-2	F: CACCGCCCCTTGCCGTCCCAAGCAA<br>R: AAACTTGCTTGGGACGGCAAGGGGC	CCCCTTGCCGTCC<br>CAAGCAATGG	NM_001126114.2
sgTP53-3	F: CACCGGGGCAGCTACGGTTTCCGTC<br>R: AAACGACGGAAACCGTAGCTGCCCC	GGGCAGCTACGGT<br>TTCCGTCTGG	NM_001126114.2
sgTP53-4	F: CACCGGGCAGCTACGGTTTCCGTCT<br>R: AAACAGACGGAAACCGTAGCTGCCC	GGCAGCTACGGTT<br>TCCGTCTGGG	NM_001126114.2
LentiCRIS<br>PRv2-<br>Screen	F: GTACCGAGGGCCTATTTCCC<br>R: CTCCTTTCAAGACCTAGCTAGCG		

Supplemental Table 6. Genotyping primers (related to Figures 7 and 8)

Mouse strain	Cre	Internal control
Sox10-Cre	F: CACCTAGGGTCTGGCATGTG<br>R: AGGCAAATTTTGGTGTACGG	F: GACAAAATGGTGAAGGTCGG<br>R: CAAAGGCGGAGTTACCAGAG
Sox10-<br>*CreERT2*	F: CACCTAGGGTCTGGCATGT<br>R: CAGGTTTTGGTGCACAGTCA	F: CAAATGTTGCTTGTCTGGTG<br>R: GTCAGTCGAGTGCACAGTTT
Nf1 flox	AATGTGAAATTGGTGTCGAGTAAGGTAACCAC	
	TTAAGAGCATCTGCTGCTCTTAGAGGGAA	
	TCAGACTGATTGTTGTACCTGATGGTTGTACC	
LacZ	F: ATTGTCAGACATGTATACCCCGTACGTCTT<br>R: TTTTGACACCAGACCAACTGGTAATGGTAG	

Supplemental Table 7. List of SOX10-CreERT2 mice (related to Figure 7 and 8)

Genotype	Sex	pNF	cNF	Life span (days)
Sox10-CreERT2; Nf1$^{f/f}$	M	Y	Y	575
Sox10-CreERT2; Nf1$^{f/f}$	M	Y	Y	518
Sox10-CreERT2; Nf1$^{f/f}$	F	Y	Y	471
Sox10-CreERT2; Nf1$^{f/f}$	M	Y	Y	488
Sox10-CreERT2; Nf1$^{f/f}$	F	Y	Y	403
Sox10-CreERT2; Nf1$^{f/f}$	F	N	Y	548
Sox10-CreERT2; Nf1$^{f/f}$	M	Y	Y	509
Sox10-CreERT2; Nf1$^{f/f}$	F	Y	Y	457
Sox10-CreERT2; Nf1$^{f/f}$	M	Y	Y	489
Sox10-CreERT2; Nf1$^{f/f}$	F	Y	Y	546
Sox10-CreERT2; Nf1$^{f/f}$	M	Y	Y	214
Sox10-CreERT2; Nf1$^{f/f}$	F	Y	Y	392
Sox10-CreERT2; Nf1$^{f/f}$	M	Y	Y	495
Sox10-CreERT2; Nf1$^{f/f}$	M	Y	Y	411
Sox10-CreERT2; Nf1$^{f/f}$	M	Y	Y	560

Sox10-CreERT2; Nf1$^{f/f}$	M	Y	N	247
Sox10-CreERT2; Nf1$^{f/f}$	M	Y	Y	247
Sox10-CreERT2; Nf1$^{f/f}$	M	Y	Y	335
Sox10-CreERT2; Nf1$^{f/f}$	F	Y	Y	470
Sox10-CreERT2; Nf1$^{f/f}$	F	Y	Y	470
Sox10-CreERT2; Nf1$^{f/f}$	M	Y	Y	401
Sox10-CreERT2; Nf1$^{f/f}$	F	Y	Y	326
Sox10-CreERT2; Nf1$^{f/f}$	F	Y	Y	412
Sox10-CreERT2; Nf1$^{f/f}$	F	Y	Y	548
Sox10-CreERT2; Nf1$^{f/f}$	M	Y	Y	350
Sox10-CreERT2; Nf1$^{f/-}$	F	Y	Y	451
Sox10-CreERT2; Nf1$^{f/-}$	F	Y	Y	411
Sox10-CreERT2; Nf1$^{f/-}$	F	Y	N	199
Sox10-CreERT2; Nf1$^{f/-}$	M	Y	N	205
Sox10-CreERT2; Nf1$^{f/-}$	F	Y	Y	263
Sox10-CreERT2; Nf1$^{f/-}$	M	Y	Y	304
Sox10-CreERT2; Nf1$^{f/-}$	F	Y	Y	304
Sox10-CreERT2; Nf1$^{f/-}$	F	Y	N	190
Sox10-CreERT2; Nf1$^{f/-}$	F	Y	Y	475
Sox10-CreERT2; Nf1$^{f/-}$	M	Y	Y	190
Sox10-CreERT2; Nf1$^{f/-}$	M	Y	N	314
Sox10-CreERT2; Nf1$^{f/-}$	M	Y	Y	418
Sox10-CreERT2; Nf1$^{f/-}$	F	Y	Y	418
Sox10-CreERT2; Nf1$^{f/-}$	F	Y	Y	418
Sox10-CreERT2; Nf1$^{f/-}$	M	Y	N	399
Sox10-CreERT2; Nf1$^{f/-}$	F	Y	Y	420
Sox10-CreERT2; Nf1$^{f/-}$	F	Y	Y	337
Sox10-CreERT2; Nf1$^{f/-}$	F	Y	Y	200
Sox10-CreERT2; Nf1$^{f/-}$	F	Y	Y	184

F: female; M: male; Y: yes; N: no.
